# Exercise as therapy for neurodevelopmental and cognitive dysfunction in people with a Fontan circulation: A narrative review

**DOI:** 10.3389/fped.2023.1111785

**Published:** 2023-02-13

**Authors:** Charlotte Elizabeth Verrall, Derek Lee Tran, Joseph Yuan-Mou Yang, David Revalds Lubans, David Scott Winlaw, Julian Ayer, David Celermajer, Rachael Cordina

**Affiliations:** ^1^ Heart Centre for Children, The Children's Hospital at Westmead, Sydney, NSW, Australia; ^2^ Children’s Hospital at Westmead Clinical School, Faculty of Medicine and Health, University of Sydney, Sydney, NSW, Australia; ^3^ Department of Cardiology, Royal Prince Alfred Hospital, Sydney, NSW, Australia; ^4^ Central Clinical School, The University of Sydney School of Medicine, Sydney, NSW, Australia; ^5^ Charles Perkins Centre, Heart Research Institute, Sydney, NSW, Australia; ^6^ Developmental Imaging, Murdoch Children's Research Institute, Melbourne, VIC, Australia; ^7^ Neuroscience Research, Murdoch Children's Research Institute, Melbourne, VIC, Australia; ^8^ Department of Paediatrics, University of Melbourne, Melbourne, VIC, Australia; ^9^ Department of Neurosurgery, Neuroscience Advanced Clinical Imaging Service (NACIS), Royal Children's Hospital, Melbourne, VIC, Australia; ^10^ Centre for Active Living and Learning, College of Human and Social Futures, University of Newcastle, Callaghan, NSW, Australia; ^11^ Hunter Medical Research Institute, New Lambton Heights, NSW, Australia; ^12^ Faculty of Sport and Health Sciences, University of Jyväskylä, Jyväskylä, Finland; ^13^ Cardiothoracic Surgery, the Heart Institute, Cincinnati Children's Hospital Medical Center, Cincinnati, OH, United States; ^14^ Heart Research Group, Murdoch Children's Research Institute, Melbourne, VIC, Australia

**Keywords:** Fontan, exercise, neurodevelopment, cognition, brain injury, intervention

## Abstract

People with a Fontan circulation are at risk of neurodevelopmental delay and disability, and cognitive dysfunction, that has significant implications for academic and occupational attainment, psychosocial functioning, and overall quality of life. Interventions for improving these outcomes are lacking. This review article discusses current intervention practices and explores the evidence supporting exercise as a potential intervention for improving cognitive functioning in people living with a Fontan circulation. Proposed pathophysiological mechanisms underpinning these associations are discussed in the context of Fontan physiology and avenues for future research are recommended.

## Introduction

**Figure 1:**
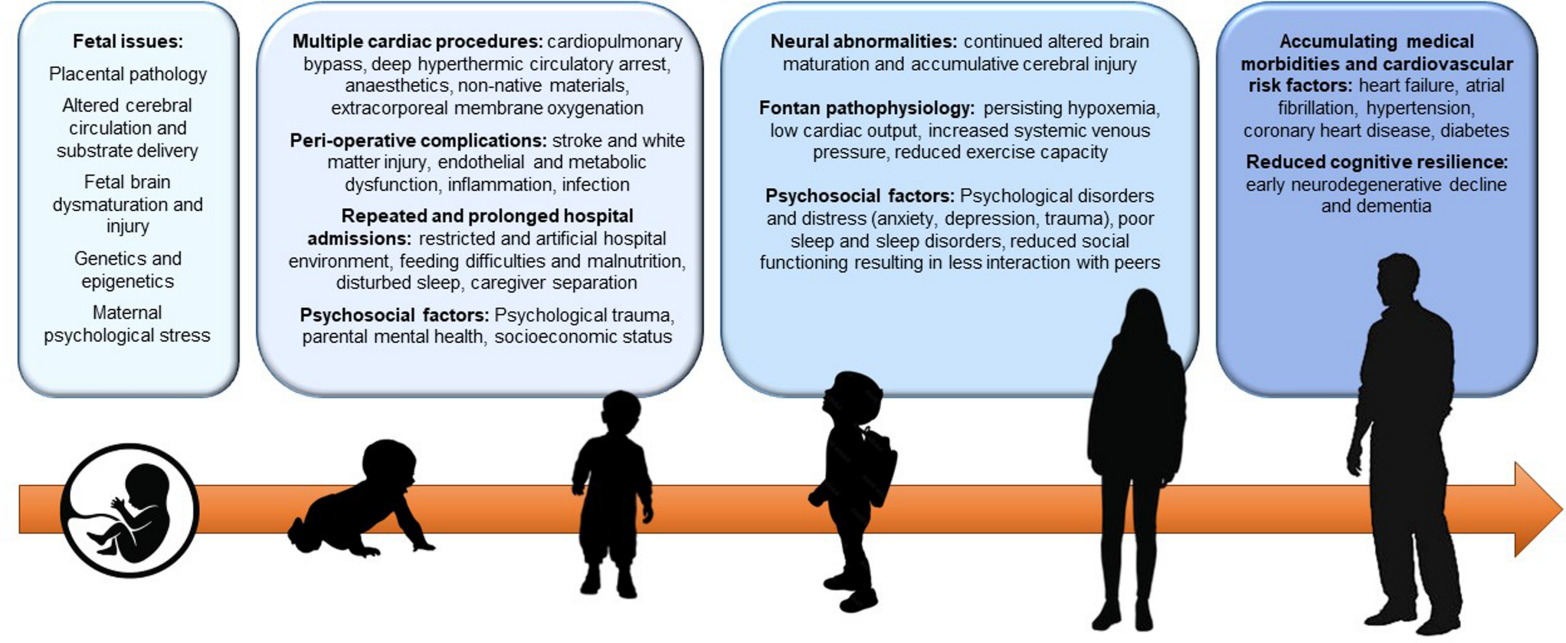
Accumulative neurodevelopmental and cognitive risk factors across the lifespan.

Neurodevelopmental and cognitive deficits are recognised as the most common comorbidities associated with complex congenital heart disease (CHD) (1). Individuals born with single ventricle heart disease, typically resulting in the Fontan circulation, are at the greatest risk of cognitive dysfunction (2–5). The complex Fontan anatomy is associated with neurological vulnerability and injury, that accumulates over the lifetime (Figure 1). Emerging research suggests that early neurodegenerative decline and dementia may also be an impending issue in this newly aging population (2, 6). These issues compound the spectrum of medical and health challenges impacting individuals with a Fontan circulation, and contribute to reduced quality of life, restricted educational and occupational achievements and increased mental health issues (4, 7).

The implementation of routine neurodevelopmental and cognitive screening and increased accessibility to neuropsychological assessment is vital for many individuals with a Fontan circulation, however, a coexisting and paramount problem is the lack of resources and evidence-based intervention strategies for those identified with deficits. While this issue is not entirely unique to the CHD population, additional considerations may be required in the development of safe and effective intervention strategies for people with Fontan physiology. Interventions that optimise outcomes beyond improved cognitive abilities would be a major advantage.

**Figure 2:**
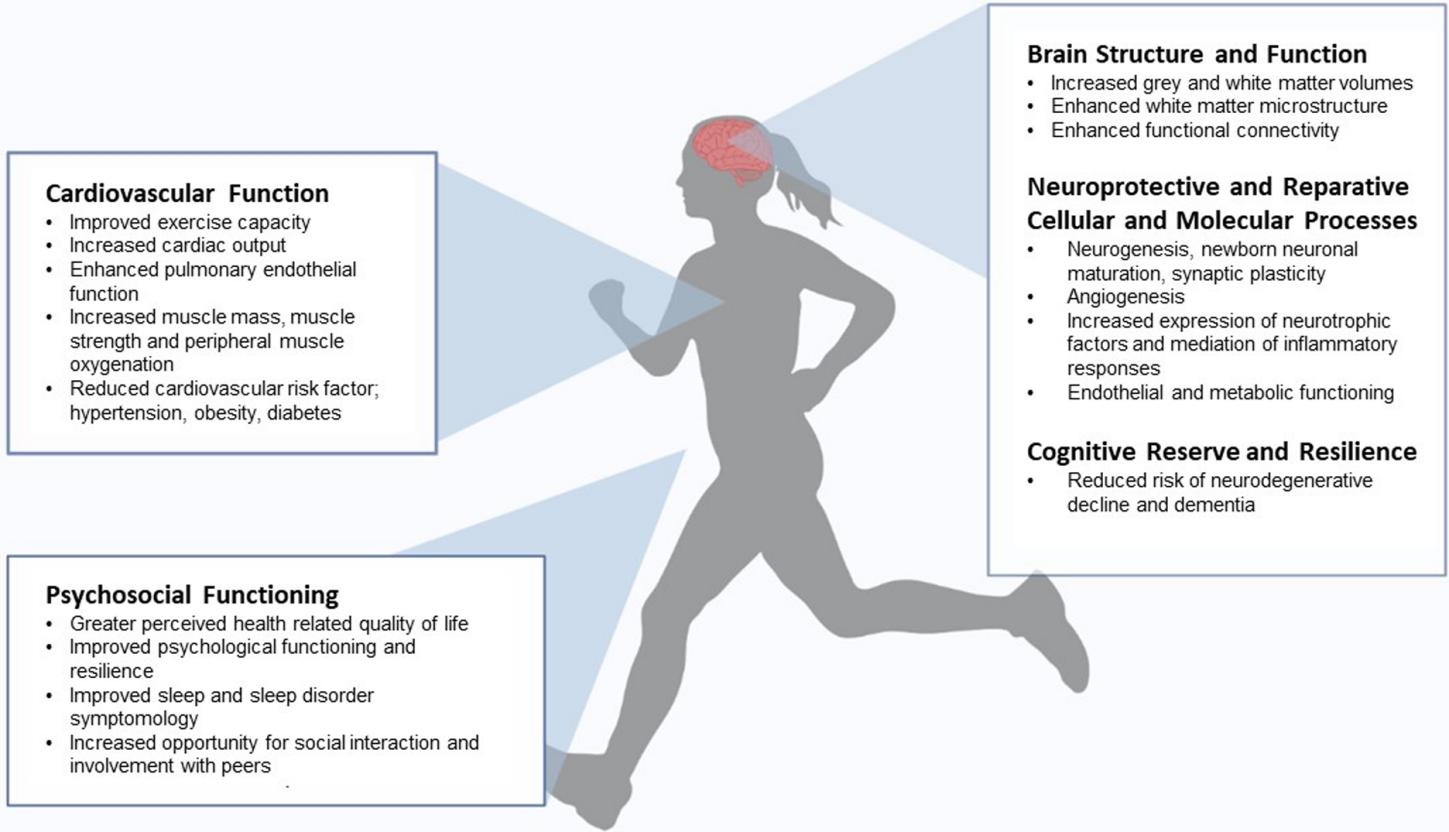
Multi-level factors associated with exercise and improved cognitive functioning.

For the purpose of this review, we focus on exercise as a proposed intervention strategy following Fontan completion. While the distinction between early neurodevelopmental disability and longer-term cognitive dysfunction is blurred in this cohort, who have a lifetime prevalence of risk factors that contribute to reduced functioning, we refer to alterations in cognitive functioning in response to exercise, acknowledging that this is not mutually exclusive to early neurodevelopmental outcomes. Proposed mechanisms underpinning the exercise-cognition relationship are summarised in Figure 2. While this review specifically focuses on individuals with a Fontan circulation, the discussion may have broad relevance to people living with other types of complex CHD.

## Established neurodevelopmental and cognitive interventions

Hospital-based interventions that target early infant neurodevelopment in babies born with a functional single ventricle are vital for optimising the developmental trajectory. While significant brain abnormality and injury occur perinatally and are associated with patient-specific factors, advances in medical and surgical strategies have optimised cerebral protection and outcomes (8). Individualised neurodevelopmental follow-up programs including formal evaluation, environmental modifications, and therapeutic intervention, have been implemented into routine clinical practice in some paediatric cardiac intensive care settings, however, are not currently standardised care (9, 10). Longer-term neurodevelopmental follow-up programs existing as part of routine cardiac care are even less common. Individually tailored developmental care plans have been shown to contribute to improved neurodevelopmental outcomes in children with CHD (11). Where neurodevelopmental delay or disability is identified, traditional interventions may typically include occupational therapy, speech pathology, and/or physiotherapy, that are often initiated within the inpatient environment. Parent-focused psychoeducation is associated with improved mental, social, and emotional development in children with CHD at 6-months of age and is associated with better family functioning and less school days missed in early childhood (11). Family-focused education should form an integral component of neurodevelopmental care for children with CHD (12).

Beyond the neurodevelopmental interventions implemented in early life, interventions may typically occur in the school setting and are predominantly focused on improving learning outcomes and academic achievement. Classroom strategies may include assisted learning and tutoring, behavioural therapies, or compensatory strategies, such as additional time to complete tasks. However, in our clinical experience, many children with a Fontan circulation who demonstrate milder cognitive difficulties, such as inattention (which is particularly common) (13, 14), often “slip through the cracks” and their learning challenges are not always recognised due to a lack of formal assessment. Where attention deficit hyperactivity disorder (ADHD) is diagnosed, stimulant medication remains the frontline treatment for children over 6 years of age and is generally considered effective and safe for people with CHD. However, the choice of stimulant medication should be guided by cardiovascular risk factors on an individual basis and appropriate monitoring is required (15). Multifactorial intervention models incorporating stimulant and behavioural intervention are considered more effective than pharmacological intervention alone (16). Exercise has been shown to be particularly beneficial in people with ADHD; Notably, moderate-to-large positive effects have been identified between exercise and a broad range of executive functions in child and adult ADHD cohorts (17–20). Exercise may be an effective alternative treatment for people with ADHD who have contraindication or opposition to commence pharmacological intervention, or where stimulant medication has been ineffective (21). Psychotherapy or the use of anti-depressants to improve low mood or anxiety may have secondary benefits for cognitive ability (22, 23), however findings are mixed and may depend on the severity of psychological symptoms and the type of treatment (24).

Cognitive remediation strategies typically utilised in adult cohorts include compensatory behavioural and environmental modifications, “brain-training” strategies, or medication when cognitive dysfunction is associated with a psychiatric or neurological condition. As the adult Fontan population continues to increase rapidly, so does the need for longer term cognitive interventions that take into account the unique and lifelong medical and psychosocial complexities associated with Fontan physiology.

The only prospective, randomised control intervention trial including individuals with a Fontan circulation investigated the benefits of computerised working memory training on executive functioning and social outcomes in people with complex CHD. Benefits in self-regulatory control were identified. However, working memory, that was the primary outcome measure, did not significantly change post-intervention. Possible long-term benefits to other cognitive domains are not yet established (25). The effectiveness of computerised cognitive training programs remains widely debated.

## Exercise, cognition, and Fontan physiology

Historically, exercise participation was discouraged for people with a Fontan circulation due to the perceived risks associated with their complex cardiac physiology, including haemodynamic derangement and sudden cardiac death. Emerging research has demonstrated that increased physical activity may not only be beneficial, but of great importance for individuals living with a Fontan circulation (26, 27). Reduced exercise capacity in this cohort is associated with worse long-term prognosis (28, 29). Exercise training has been shown to positively affect stroke volume, cardiac output, lung function, and skeletal muscle mass, subsequently optimising overall physical function and exercise capacity (30–34). Exercise participation is now strongly encouraged as a safe therapy for most children and adults with Fontan physiology (35, 36), although guidance regarding the nature and intensity of exercise should continue to be provided to patients on an individualised basis (37).

In healthy children and adults, greater exercise capacity and physical fitness are associated with better cognitive functioning (38–40) and physical inactivity is a risk factor for cognitive impairment (41, 42). Longitudinal studies have shown improved cognitive functioning after aerobic and/or resistance exercise training programs (43). Notably, improvements in executive functioning associated with increased physical fitness and greater exercise participation have been widely reported in both children and adults (44–46), which is a key area of dysfunction in people with a Fontan circulation (2, 47). Greater exercise capacity and physical activity may have a neuroprotective role by promoting cognitive resilience and preventing neurodegenerative decline and dementia (42). This is an important consideration for individuals with a Fontan circulation, who are predicted to be at increased risk of reduced cognitive reserve and early-onset dementia (6). Emerging research has shown significant associations between exercise capacity and sustained attention and adaptive functioning in people with a Fontan circulation (48). The extent to which exercise capacity may improve other cognitive functions (e.g., working memory, processing speed, executive function and/or internalising symptoms) were undetermined in this cohort, that was limited by modest sample sizes. Self-reported improvements in cognitive functioning have also been observed in children and young adults with a Fontan circulation and tetralogy of Fallot following a standardised 12-week aerobic exercise program (49). However, to our knowledge, no other studies have investigated the effect of exercise on cognitive performance among individuals with a Fontan circulation, or other CHD cohorts.

## Neural mechanisms underpinning the exercise-cognition relationship

### Structural morphology: brain volumes and white matter microstructure

The pathophysiological mechanisms underpinning associations between exercise and cognitive functioning are not fully understood. Neuroimaging studies have demonstrated that improved cognitive function associated with exercise capacity and physical activity is paralleled by differences in brain structure, connectivity, and functioning (45). Associations between brain morphology and cognitive functioning in people living with a Fontan circulation are also incompletely understood, however smaller brain volumes are considered a key structural underpin associated with worse cognitive outcomes (2, 50, 51). Significant associations between exercise and brain volumes have been observed in healthy aging adults. Greater levels of physical activity have been associated with larger total brain and white matter volume (52–54) and hippocampal volume (55). Notable associations are reported between cardiorespiratory fitness and grey matter volumes in regions that typically show the greatest age-related volume loss, including frontal, superior parietal, and temporal cortices, and the hippocampus (56–58). These are regions that have associated roles in executive functioning and memory decline. Taken together, these findings suggest that exercise capacity and physical activity may attenuate progressive brain volume loss associated with aging, that appears to be accelerated in adults living with a Fontan circulation (2). However, future research is necessary to determine causal associations. Other factors such as gender, comorbidities, and interindividual variation are also likely to influence the relationship between exercise capacity and brain structure (59–61).

Significant associations between hippocampal volume, fitness, and memory performance have been demonstrated in healthy pre-adolescent children (62), and an emerging study by Valkenborghs and colleagues has identified changes in hippocampal metabolism and working memory in response to a 6-month exercise intervention program in adolescents (63); Suggesting that exercise may enhance hippocampal development and structure in the developing brain, as well as possibly mitigate age-related volume loss in the aging adult brain. In infants and young children with a Fontan circulation, smaller brain volumes are predominantly attributed to cerebral dysmaturation that is associated with altered cerebral perfusion and blood flow, placental insufficiency, and genetic vulnerability (3, 64). Associated perioperative white matter injury is a major concern (65). Early interventions to mitigate these factors remain experimental but are vital for promoting early neurodevelopment, however the extent that perinatal brain dysmaturation can be modified remains unknown. These early interventions are not the focus of the current review and have been discussed in detail elsewhere (3). Exercise may be a beneficial therapeutic strategy beyond the unstable surgical period to enhance brain volumes and mitigate the impact of early brain injury by promoting neurogenesis and synaptic plasticity, which has been shown to continue in certain brain regions throughout the human lifespan, notably in the hippocampal region (66, 67). Extensive animal studies have shown that exercise promotes repair processes, including enhanced neurogenesis, new-born neuron maturation and increased functional plasticity in the hippocampus of rodents (68); These alterations are likely to be associated with improved learning and memory and mood regulating processes (69). In the first controlled intervention study of its kind, Riggs et al. (2017) demonstrated that aerobic exercise promoted hippocampal growth and improved the white matter architecture of various white matter tracts in children who had been treated with radiation therapy for a brain tumour. White matter damage and smaller bilateral hippocampal volume were present prior to the intervention, and changes in fractional anisotropy and hippocampal volume were observed irrespective of typical age-related brain maturational changes—These findings suggest that exercise may have the potential to promote neural recovery during childhood (70). We can only speculate on these associations in the context of Fontan physiology, especially given that the mechanisms of cerebral injury vary, however these findings warrant investigation. Hippocampal volume is significantly reduced in adolescents with a Fontan circulation and is associated with worse memory performance (50). Widespread alterations in white matter microstructure are reported across the lifespan in people with a Fontan circulation and have shown variable associations with cognitive performance (71–75). Cross-sectional studies in healthy child and adult cohorts support associations between exercise and enhanced white matter microstructural architecture (76, 77), however associations in other clinical cohorts are scarce and the extent that exercise-induced changes in white matter microstructure extends to improvements in cognitive functioning is undetermined (78). Exploratory work by our research team demonstrated a significant positive association between predicted peak oxygen uptake during exercise (an indicator of aerobic fitness) and fractional anisotropy of the uncinate fasciculus in an adolescent and adult Fontan cohort (75), suggesting the association between exercise capacity and white matter microstructure may apply to this cohort. Predovan et al. (2021) similarly found increased fractional anisotropy of the uncinate fasciculus associated with increased aerobic exercise in healthy adults following a 6-month randomised exercise intervention program, although significance was lost after correction for multiple comparisons (79). These findings were inconsistent in another similar study (80). An issue across diffusion MRI research surrounds the heterogeneity in methodologies utilised and continual advances are being made in the recommended diffusion data acquisition, processing methods, and analysis pipelines (81). Further research is necessary to determine associations between exercise and white matter architecture and consistent methodologies are required when investigating the replicability of findings across cohorts.

Our previous work demonstrated significant positive associations between resting and/or peak exercise oxygen saturations and brain volumes; white matter microstructural organisation was also worse in the setting of reduced oxygen saturations (2, 75). Low oxygen saturations may impair exercise performance due to reduced oxygen delivery to the peripheral muscles (34, 82), although the extent to which this is a causative factor remains undetermined (26). Nevertheless, efforts to maximise oxygen saturations in people with Fontan physiology may be important to consider for optimising both exercise capacity and brain morphology.

### Cellular, molecular and vascular processes

Exercise may have a potential role in protecting the brain against the persisting and accumulative burden of hypoxic-ischaemic events, that is an ongoing concern throughout life for people living with a Fontan circulation (2, 64). Animal studies have demonstrated that exercise training preconditions brain ischaemic tolerance and facilitates functional recovery following brain injury through a series of cellular and molecular mechanisms, including the promotion of angiogenesis, mediation of the inflammatory response, inhibition of gamma aminobutyric acid (GABA), protection of the blood brain barrier, and inhibition of apoptosis (83), suggesting that patients with high risk for chronic brain injury should engage in regular exercise to promote neuroprotection. Exercise is associated with increased expression of brain derived neurotrophic factor (BDNF) and insulin-like growth factor-1 (IGF-1), that are directly involved in neuronal and synaptic growth, and angiogenesis. In humans, BDNF and IGF-1 have been shown to mediate improvements in executive function after a 12-month aerobic exercise intervention program (84) and are considered crucial for long-term memory functioning (83). Furthermore, increased BDNF concentrations are observable following acute exercise participation and are associated with immediate improvements in processing speed, executive functioning, learning speed, and memory after high intensity training, suggesting that exercise may induce acute benefits on cognitive functioning and daily participation in exercise may bolster day-to-day cognitive functioning (85, 86). Increased expression of neurotrophic factors may also protect against neurodegenerative decline (83).

Skeletal muscle mass may be an important mediator in the relationship between exercise and neuroprotective mechanisms. Skeletal muscle secretes circulating myokines in response to exercise, that have a role in molecular and cellular neuroprotective processes in the brain, including the expression and regulation of BDNF, among others (87–89). Indeed, significant associations have been identified between sarcopenia and cognitive decline in healthy aging cohorts (90–92). Fontan-associated myopenia is a recently established phenomena (93) and may contribute to the myriad of risk factors associated with Fontan physiology and reduced cognitive functioning.

Emerging research also highlights the role of the cerebral endothelium in the release and synthesis of BDNF, that is considered to be an important link between endothelial function and cognition. Other important processes associated with endothelial function, include the regulation of inflammatory and immune responses, thrombosis, neuroplasticity, angiogenesis, and cerebral blood flow (94, 95). Endothelial dysfunction is common in people living with a Fontan circulation and is associated with reduced exercise capacity and health related quality of life (96). In other cardiovascular disease cohorts, endothelial dysfunction is linked to cerebrovascular damage including white matter hyperintensities, lacunar infarct, brain atrophy, and cerebral hypoperfusion (97–99). In adults with cyanotic CHD, endothelial dysfunction has been associated with reduced global grey matter volume (100), however associations with neurological outcomes in a Fontan cohort are yet to be examined. Markers of endothelial dysfunction are associated with worse performance in various cognitive domains, including processing speed, attention, executive function, memory, and visuospatial abilities (101–104). Endothelial dysfunction may be an early predictor of neurodegenerative disease (105). Aerobic and resistance exercise training have independently been shown to improve endothelial function in healthy individuals and those with cardiovascular disease (106–108), providing further evidence to support the relationship between exercise and brain health. Improvement in endothelial function following a 3-month exercise intervention has been associated with improved overall cognition in individuals with coronary artery disease (109). However, to our knowledge, no randomised controlled studies exist investigating these associations and causative links are undetermined. Non-invasive markers of endothelial dysfunction provide an accessible opportunity to investigate these factors. Future longitudinal and cross-sectional studies are encouraged to include such measures when investigating associations between exercise and neurological and cognitive outcomes in people with Fontan physiology.

### Functional brain activation in response to exercise

Less is known about functional brain changes occurring in response to exercise, however functional MRI (fMRI) research using Blood Oxygen Level Dependent (BOLD) signal as indirect markers of neuronal activities has provided additional support for the association between vascular risk factors and cognitive dysfunction (110). Changes in BOLD functional brain activation are observed following acute bouts of physical exercise, and sustained exercise over an 8-month period has been shown to improve spatial refinement of several key functional brain networks at rest (111). However, the associated changes in cognitive functioning with exercise-induced functional brain activation are variable and limited research investigating these associations has been conducted to date (112, 113). Patterns of exercise-induced functional brain activation also vary across different cohorts and may be influenced by levels of cardiorespiratory fitness (113). Studies including individuals with a Fontan circulation are required to understand these associations in the context of Fontan physiology and will provide insight into functional brain connectivity in this cohort. Even less is known about exercise-induced changes in neurotransmitter release and associated cognitive performance.

## Psychosocial and behavioural mediators of the exercise-cognition relationship

### Psychological functioning

It is likely that improved behavioural and psychosocial functioning associated with exercise participation and greater fitness, also mediate improvements in cognitive functioning. Children and adults with a Fontan circulation are at an increased risk of psychological distress and developing psychiatric disorders, that is associated with a myriad of factors linked to their cardiac anatomy (114). Physical restrictions and reduced exercise capacity likely contribute to these issues. The lifetime prevalence of a psychiatric disorder in people living with a Fontan circulation has been reported as 65% and includes a high incidence of depression, anxiety, and medically related trauma, reduced psychosocial functioning is also prevalent (4, 115, 116). People with major depressive disorder and high levels of anxiety demonstrate reduced attention, executive functioning, and memory (117), that are cognitive domains similarly most affected by physical inactivity (118). The rate of comorbidity between psychological disorders and cognitive dysfunction in people living with a Fontan circulation is anticipated to be high but has not yet been investigated.

The relationship between exercise and improved mental health and overall quality of life is well-established and broadly applies to clinical and healthy populations (119–123). Furthermore, participation in physical activity and sport is considered to improve social health and functioning through increased opportunities to socialise and connect with peers (124), that may also provide opportunities to practice behavioural and emotional self-regulation. In children and young adults with a Fontan circulation, exercise training is associated with improved health related quality of life and psychosocial functioning (49, 125, 126). These benefits are anticipated to extend to better family functioning and improved quality of life for caregivers and siblings, who are also importantly impacted (127, 128). Important associations have also been identified between physical inactivity and greater rates of depression in people with various forms of congenital heart disease (129). No studies have yet directly investigated the link between exercise, psychosocial functioning, and cognitive outcomes in people with a Fontan circulation. Importantly, interventions to promote psychological functioning, independent of cognitive dysfunction, are also greatly needed in this cohort (114).

### Sleep

Sleep disturbance and fatigue are also important risk factors that may contribute to reduced cognitive functioning and are commonly reported by people with a Fontan circulation. In a large study of children with a Fontan circulation, self- and parent-reported sleep disturbance was strongly associated with increased odds of attentional problems, the use of medication for ADHD, behavioural issues, developmental delay, learning problems, mood concerns, and reduced health related quality of life (130). Hedlund et al. (2019) have demonstrated positive associations between total sleep time and time engaged in moderate-vigorous physical activity in children and adolescents with a Fontan circulation (131). Patients and controls with low sleep efficiency (amount of time in bed spent sleeping) at baseline demonstrated increased sleep efficiency following exercise endurance training. Consistent with this, Callegari et al. (2022) found a significant association between daily minutes of moderate-vigorous physical activity and a lower incidence of sleep disturbance in a similar Fontan cohort (132). Sleep efficiency has been shown to mediate the relationship between physical activity and improved executive functioning in healthy young and older adults (133). In contrast, total sleep duration does not always show consistent associations as this may be impacted by sleep interruptions that interfere with important sleep cycle stages that are associated with restorative pre-frontal and hippocampal functioning (134). Excessive sleep may also reflect depressed mood or chronic fatigue. Increased exercise and improved cardiorespiratory fitness may also alleviate symptoms of sleep disordered breathing, that is a recognised concern in children and adults with a Fontan circulation (135–137), and may be a modifiable contributor to reduced cognitive performance (138).

## Uncertainties and future directions

Exercise training is garnering much enthusiasm as a possible non-pharmacological therapy for a range of physical, psychological, and cognitive illnesses. However, the mechanisms by which exercise exerts benefit on cognitive outcomes are not fully understood and are likely multifactorial and influenced by interindividual differences. In addition, methods to investigate cellular and molecular neural changes *in-vivo* are limited, and much of what we currently know has come from animal studies that may have limited applicability. Well-designed and adequately powered, randomised controlled human intervention studies are critically needed to investigate these associations in the context of Fontan physiology and detailed phenotyping is required to understand possible mediating factors. Where feasible, minimally invasive methods to directly, or indirectly, investigate underlying mechanisms are required. Animal models with Fontan physiology may provide additional avenues for future research in this regard.

The type, frequency, and intensity of regular exercise are likely important factors for facilitating cognitive change, and overall, there appears to be a dose-response relationship between exercise and cognitive performance (118, 139). Moderate to vigorous exercise shows the most consistent benefit on cognitive performance, however some studies have found that participation in low intensity exercise, such as walking, yoga, and balance or resistance training also improves cognitive outcomes (140–143), which may be promising for patients with more severe exercise limitation. Different types of exercise appear to be associated with different underlying neural mechanisms associated with cognitive outcomes (144). Aerobic exercise is predominantly associated with the production and regulation of BDNF, whereas resistance training is associated with increased expression of IGF-1 (145, 146). Combined training programs incorporating both aerobic and resistance training are therefore considered optimal to target varying molecular pathways that have a beneficial impact on brain health. Better understanding the detailed mechanisms by which exercise improves cognitive outcomes may provide an opportunity to develop novel therapeutic interventions that mimic these mechanisms in individuals with Fontan physiology who are unable to exercise.

Exercise interventions introduced early in childhood will likely yield maximum benefits for people with a Fontan circulation, both physically and cognitively. Engagement in exercise as a child is associated with continued exercise participation in adulthood, which is anticipated to maximise cognitive resilience for aging adults with a Fontan circulation. The long-term benefits of exercise interventions programs remain largely undetermined, and it is likely that regular and consistent exercise participation is required to maintain optimum cognitive functioning.

While current recommendations highlight the importance of exercise for people living with a Fontan circulation (26, 34, 37, 147), the optimal methods and guidelines surrounding exercise prescription remain poorly characterised. The primary target of exercise intervention in this cohort is to optimise cardiac and physical functioning, however investigations should consider the intensity, duration and type of exercise that is required to also maximise cognitive outcomes. Additional efforts will likely be required to facilitate long-term behaviour change surrounding exercise participation in many people with a Fontan circulation, given the multitude of factors that contribute to reduced physical activity in a large proportion of the population.

### The Fontan-Fitness Intervention Trial (F-FIT)

To our knowledge, the Fontan-Fitness Intervention Trial (F-FIT) (26) will be the first multicentre, randomised controlled exercise intervention trial investigating the impact of aerobic and resistance exercise training on physiological and cognitive outcomes in people with a Fontan circulation. Participants will be recruited across eight quaternary CHD centres in Australia and will include people with a Fontan circulation aged 10–55 years, who are a minimum of 6 months post-Fontan completion; broader inclusion and exclusion criteria are described by Tran et al. (2022). The protocol includes a range of outcome measures that aims to contribute to our knowledge surrounding the associations and risk factors contributing to cardiorespiratory fitness, cognitive functioning, psychosocial outcomes, and overall quality of life in people with Fontan physiology. This includes a detailed evaluation of aerobic exercise capacity, respiratory muscle and lung function, musculoskeletal fitness, body composition, habitual physical activity levels, dietary intake and nutritional status, peripheral venous pressure, endothelial function, neurohormonal activation, metabolites, cardiac function, cognitive and psychosocial functioning, quality of life, and sleep. A subset of participants with Fontan physiology will undergo neuroimaging, including structural and diffusion weighted brain MRI and measurement of cerebral blood flow.

For adolescents and adults (≥16 years), the protocol includes a three-arm blinded randomisation to either (i) traditional fitness facility-based exercise training, (ii) telehealth exercise training, or (iii) waitlist control. Investigation into the efficacy of a telehealth-based cognitive intervention is a key advantage of the F-FIT protocol. Socioeconomic status and geographic remoteness are associated with worse cognitive outcomes in adolescents and adults living with a Fontan circulation (2) and these individuals may have additional barriers to accessing appropriate heath care. Exercise may be a cost-effective life-long therapeutic management strategy for this cohort, which is a particularly important consideration given the high financial burden associated with Fontan physiology (148).

## Conclusion

Individuals with Fontan physiology are at heightened risk of neurodevelopmental and cognitive dysfunction. Interventions focused on improving these outcomes are needed. Exercise may be a low-risk and broadly advantageous physical and cognitive intervention strategy in this cohort and may protect and optimise brain health across the life span. High quality experimental research is required and should aim to include comprehensive phenotyping and investigate optimum exercise “prescription”. Scalable and accessible interventions that also facilitate behaviour change are essential.

## Author contributions

CV reviewed all relevant literature and wrote the first draft of the manuscript. DT, JY, DL, DW, JA, DC and RC provided clinical expertise and relevant edits to the manuscript. All authors contributed to manuscript revision, read, and approved submitted version.
